# Sapporo: A workflow execution service that encourages the reuse of workflows in various languages in bioinformatics

**DOI:** 10.12688/f1000research.122924.2

**Published:** 2024-06-24

**Authors:** Hirotaka Suetake, Tomoya Tanjo, Manabu Ishii, Bruno P. Kinoshita, Takeshi Fujino, Tsuyoshi Hachiya, Yuichi Kodama, Takatomo Fujisawa, Osamu Ogasawara, Atsushi Shimizu, Masanori Arita, Tsukasa Fukusato, Takeo Igarashi, Tazro Ohta

**Affiliations:** 1 Department of Creative Informatics, Graduate School of Information Science and Technology, The University of Tokyo, Bunkyo, Tokyo, Japan; 2 Bioinformation and DDBJ Center, National Institute of Genetics, Mishima, Shizuoka, Japan; 3 Genome Analytics Japan Inc, Shinjuku, Tokyo, Japan; 4 Barcelona Supercomputing Center (BSC), Barcelona, Spain; 5 Curii Corporation, Sommerville, MA, USA; 6 Department of Computational Biology and Medical Sciences, Graduate School of Frontier Sciences, The University of Tokyo, Bunkyo, Tokyo, Japan; 7 Department of Computer Science, Graduate School of Information Science and Technology, The University of Tokyo, Bunkyo, Tokyo, Japan; 8 Institute for Advanced Academic Research, Chiba University, Chiba, Japan; 9 Database Center for Life Science, Joint Support-Center for Data Science Research, Research Organization of Information and Systems, Mishima, Shizuoka, Japan; 10 Department of Artificial Intelligence Medicine, Graduate School of Medicine, Chiba University, Chiba, Chiba, Japan

**Keywords:** workflow, workflow language, workflow execution service, open science

## Abstract

The increased demand for efficient computation in data analysis encourages researchers in biomedical science to use workflow systems. Workflow systems, or so-called workflow languages, are used for the description and execution of a set of data analysis steps. Workflow systems increase the productivity of researchers, specifically in fields that use high-throughput DNA sequencing applications, where scalable computation is required. As systems have improved the portability of data analysis workflows, research communities are able to share workflows to reduce the cost of building ordinary analysis procedures. However, having multiple workflow systems in a research field has resulted in the distribution of efforts across different workflow system communities. As each workflow system has its unique characteristics, it is not feasible to learn every single system in order to use publicly shared workflows. Thus, we developed Sapporo, an application to provide a unified layer of workflow execution upon the differences of various workflow systems. Sapporo has two components: an application programming interface (API) that receives the request of a workflow run and a browser-based client for the API. The API follows the Workflow Execution Service API standard proposed by the Global Alliance for Genomics and Health. The current implementation supports the execution of workflows in four languages: Common Workflow Language, Workflow Description Language, Snakemake, and Nextflow. With its extensible and scalable design, Sapporo can support the research community in utilizing valuable resources for data analysis.

## Background

Modern experimental instruments that convert biological samples into digital data have lower costs and higher throughput than conventional ones. ^1^ Those instruments have made it possible to conduct large-scale data-driven biology, not only in large projects but also in smaller studies. A DNA sequencer is one such technology in biology, which has shown a drastic improvement in throughput since the late 2000s. ^1^ DNA sequencing technology highlighted the data science aspect of biology, sparking the demand for computation in biology. ^2^

Raw data, the fragments of nucleotide sequences for a DNA sequencer, often called “reads,” are not biologically interpretable in their unprocessed form. Researchers need to process the data using computational methods to obtain biological insights from the samples. The data processing includes, for example, estimation of sequence error rates, read alignment to a reference genome sequence, extraction of genomic features from aligned data, and annotation with the information obtained from public databases. Researchers develop and share the command-line tools for each step in an analysis. They use the raw data as the initial input data of the first tool and pass its output on as input for the next tool. This chain of processes, connecting a sequence of tools according to their inputs and outputs, is called a workflow. ^3^

Workflow structure can be complicated as various sequencing applications require multiple steps of data processing. Combining many tools to construct a complex workflow that performs as intended is not straightforward. It is also not practical to fully understand the internal processes of all the tools. Thus, ensuring that every individual part of a workflow is working correctly depends heavily on the skills of the workflow developer. Even if a workflow runs successfully once, maintaining it is another issue. The tools in a workflow are often developed as open-source software and are frequently updated to improve performance and fix bugs. It is time-consuming to assess the impact of updates associated with individual tools. The tools in a workflow often work in an unintended manner for many reasons, such as changes in hardware, operating system (OS), software dependencies, or input data. Difficulties in building and maintaining workflows cause portability issues with workflows. ^4^ Because of this, researchers have to spend a great deal of time building workflows similar to those that others have already created.

To address these issues, researchers have developed many workflow systems in bioinformatics. ^5^ Each workflow system has unique characteristics, but generally, they all have a language syntax and a workflow engine. Workflow languages define a syntax to describe the inputs and arguments passed to tools and the handling of outputs. Workflow engines often take two arguments to execute a workflow: a workflow definition file that specifies the processes and a job file for input parameters. In many cases, techniques, such as package managers and container virtualization, make it easier to build, maintain, and share complex workflows by pinning down the versions of workflow tools. ^6^

Open-source workflow systems help the research community work efficiently by reusing published workflows. ^7^ However, having multiple systems has resulted in resources distributed across various workflow system communities. For example, the Galaxy community is known for being one of the largest for data analysis in biology. ^8^ The community maintains a number of workflows and learning materials that users can run on public Galaxy instances. However, as the Galaxy workflows are only runnable on the Galaxy platform, users will face difficulties in running these workflows on other platforms. As another example, Nextflow, one of the most popular command-line-based workflow systems, has a mature community called nf-core to share standard workflows. ^9^^,^^10^ The community has excellent resources, but these are usable only by Nextflow users. It is not reasonable to have a “one-size-fits-all” workflow system in science because various approaches have pros and cons. ^3^ Learning the different concepts and features of each workflow system has a high cost associated with it. Thus, it is not practical to consider becoming familiar with a large number of workflow systems in order to be able to utilize the workflows shared by their community users.

Workflow systems have different language syntaxes and engines, each designed for specific purposes. For instance, Nextflow aims to boost developer productivity and scalability, while Snakemake focuses on flexibility and simplicity, using Python as its base. In contrast, the Common Workflow Language (CWL) project aims to promote interoperability by creating a standardized syntax that various workflow engines can understand. However, workflows written in different languages cannot be easily converted into each other automatically. The most popular workflow systems used in bioinformatics, such as CWL, WDL, Nextflow, and Snakemake, take a workflow definition and input parameters to produce output result files, while there are differences between these workflow systems in command-line options, workflow description syntax, methods for specifying inputs, and how expected output files are defined.

Creating a universal language converter isn’t practical because some languages lack the necessary syntax parsers, or contain features that are not commonly found in other workflow engines (e.g. JavaScript evaluation as in CWL, loops in workflows or cyclic workflows instead of DAG-based systems). To bridge the gap between different workflow systems, we need a standardized way to specify workflows, input parameters, and expected outputs. Additionally, a system that supports various engines and selects the appropriate one for a given workflow is essential for smooth interoperability.

In this paper, we introduce Sapporo, a platform for running multiple workflow systems in the same computing environment. Sapporo wraps the differences in the workflow systems and provides an application programming interface (API) for executing them in a unified way. Sapporo also provides a graphical user interface (GUI) that works as its API client. By enabling users to run multiple workflow systems on the same computing environment, Sapporo gives users the ability to reuse workflows without having to learn a new workflow system.

## Methods

### System overview

**Figure 1.**
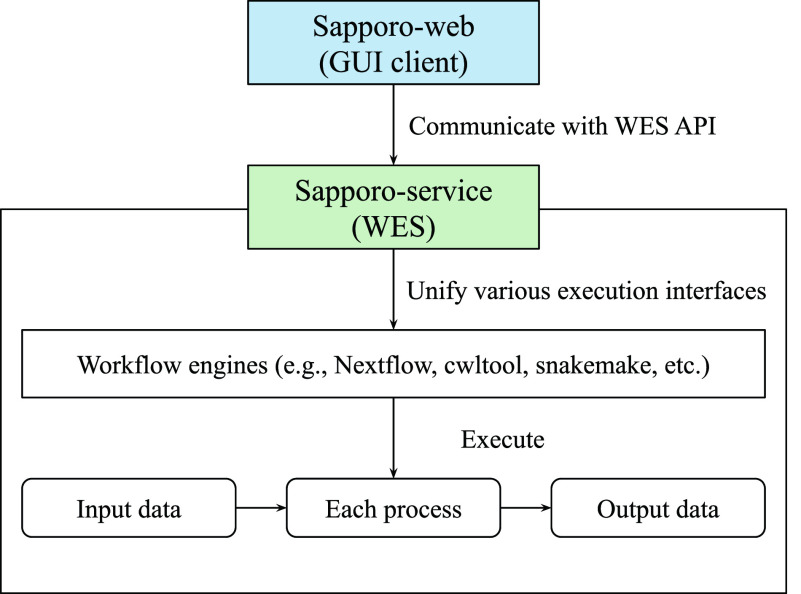
Overview of the Sapporo system. The component at the bottom is Sapporo-service, a Global Alliance for Genomics and Health (GA4GH) Workflow Execution Service (WES) standard compatible application programming interface (API) to manage the workflow execution. The box at the top is Sapporo-web, the graphical user interface (GUI) client for WES implementations. Sapporo-service has the open specification of the API endpoints, which users can access programmatically.

Sapporo consists of two components: Sapporo-service and Sapporo-web ( Figure 1). Sapporo-service is an API that receives requests for workflow execution from clients, then executes them in a specified manner. Sapporo-service has an API scheme that satisfies the Global Alliance for Genomics and Health (GA4GH) Workflow Execution Service (WES) standard. ^11^ Sapporo-web is a workflow management client. It is a client of Sapporo-service and other GA4GH WES compatible API servers. The GUI is a browser-based application that does not require user installation.

**Figure 2.**
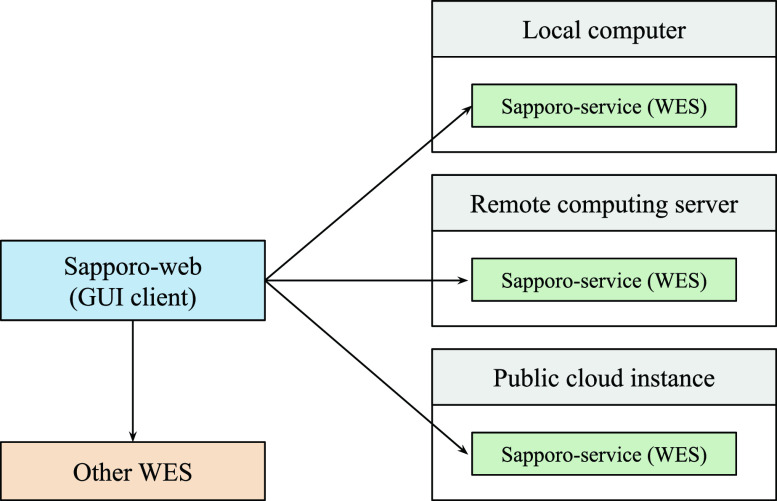
The distribution model of the Sapporo system. Researchers often have multiple computing environments for their data analysis. We designed the Sapporo system to work with a distributed computation model. For example, users can deploy the application programming interface (API) on their local computer, remote computing server, and public cloud instances. As long as the API is running, the user can send a request to execute a workflow from a local client computer.

We designed the Sapporo system based on the concept of microservices architecture. ^12^ Unlike conventional computation server applications, we expect multiple Sapporo-service instances to be run on servers as independent endpoints on demand. To manage the runs on the different API servers, we separate the implementation of the server and its client, allowing clients to connect to multiple servers ( Figure 2). One of the unique features of the Sapporo system is that it has no authentication mechanism on the application layer. Instead of having users’ information on the server-side, the user’s web browser stores the information, such as workflow execution history. The online documentation “Sapporo: Getting Started”, available in *Extended data*, shows the step-by-step procedures to deploy a Sapporo instance on a local computer to test the system. ^13^^,^^34^

The source code, test code, and documentation for Sapporo-service and Sapporo-web are available from GitHub and archived in Zenodo. ^35^^,^^36^

### Workflow execution service

**Figure 3.**
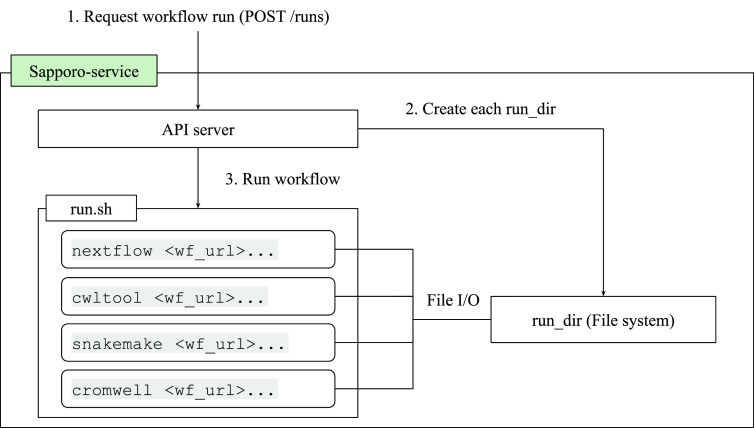
The Sapporo-service components. Sapporo-service’s application programming interface (API) layer implemented in Python works as an API server to receive the request of a workflow run. The system can be deployed easily by using Docker compose manifest provided in the GitHub repository (See Software availability). Once the API receives the request, it creates a directory to store all the related information and execute run.sh. The run.sh script receives the arguments from the API request and runs the workflow with the specified workflow engine.

The WES has two layers: the API and the execute function ( Figure 3). The API structure and the response are compliant with the GA4GH WES standard. ^14^ The API specification defines the methods to manipulate workflow runs, such as execution, stop, and checking the outputs. In addition, Sapporo-service has its own unique features ( Table 1). The key feature that makes Sapporo notable is the workflow engine selection. While the other workflow management systems accept one or a few workflow languages, Sapporo-service can accept any workflow language as long as it has a corresponding workflow engine.

**Table 1.** The list of Sapporo-service’s features.

Feature	Description
Engine selector	Select engine from available implementations
Remote URL as attachment	Fetch remote file and attach to the run
Output downloader	Direct download of workflow results
Registered-only mode	Restrict workflows by allowed-list
Workflow parser	Return parsed workflow information

**Figure 4.**
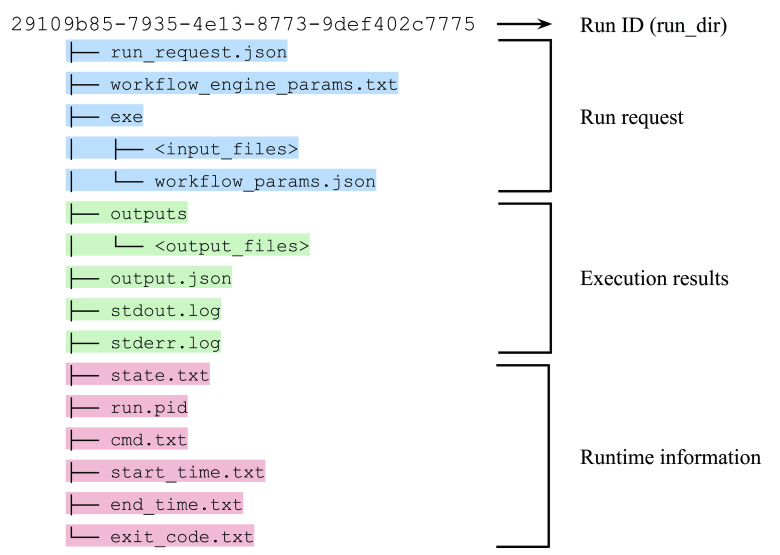
The contents in the per-run directory. Various types of information, such as run requests, execution results, and runtime information, are stored as a bundle of provenance for the workflow run.

The system is designed to separate the execution layer from the handling of API requests, thereby enhancing modularity and extensibility. The execution layer operates through a well-structured shell script named “run.sh.” Upon receiving an API request, the system forks “run.sh,” which then generates command lines for the workflow system and executes them. This separation enables the addition of new workflow systems without changes to the API server’s code. As a result, adding new workflows becomes straightforward, with the number of systems growing from just one at the beginning of the project to seven in the current version ( Table 2). The flexibility of the “run.sh” also allows for specific adjustments for each workflow system, supporting pre- and post-execution processes, such as authentication, staging input files, and uploading results. Additionally, it is enabled to manage environment-specific requirements, including executing jobs on grid engines and handling file I/O with S3-like object storage. Once the system receives a workflow run request, it issues a universally unique identifier (UUID) and creates a directory named with the UUID, where the system stores all the necessary files. The workflow definition files, intermediate and final outputs, and the other metadata are stored in that directory. This per-run directory can act as a bundle of provenance for the workflow run ( Figure 4).

**Table 2.** The list of workflow engines available in Sapporo.

Engine	Supported language(s)
cwltool ^15^	CWL
Nextflow ^9^	Nextflow
Toil ^16^	CWL
Cromwell ^17^	WDL, CWL
Snakemake ^18^	Snakemake
ep3 ^19^	CWL
StreamFlow ^20^	CWL

The system has no backend database as it stores all the information in the file system. This architecture allows the system administrators to manage the data as they do for normal server operations. We also provide a Docker image of the application, which can completely separate the system into the application (container image) and data (file system) for better portability and scalability. ^21^

Another feature not implemented in a standard WES server is a registered-only mode. By enabling it at the server start-up, users can execute only the workflows in the allowed list specified by the administrator. This function helps the administrators launch a public WES instance while preventing suspicious programs from running on the server. Instead of implementing user authentication on the application, we expect the administrators to do the required authentication to the server on the network layer, such as virtual private network (VPN).

### Workflow management console

We designed Sapporo-web as a browser-based GUI client for GA4GH WES endpoints. Sapporo-web can also be easily deployed by using the Docker compose manifest provided in the GitHub repository (See Software availability). The system is a JavaScript application that runs on a web page, which users do not need to install on their computers. It stores user data in the browser’s local storage, so users do not need to sign up to start running workflows. No information other than the access log is preserved on the server-side. The Sapporo-web system is compliant with the GA4GH WES specification. We used Elixir WES, another WES implementation, to confirm Sapporo’s GA4GH WES specification compliance. ^22^

**Figure 5.**
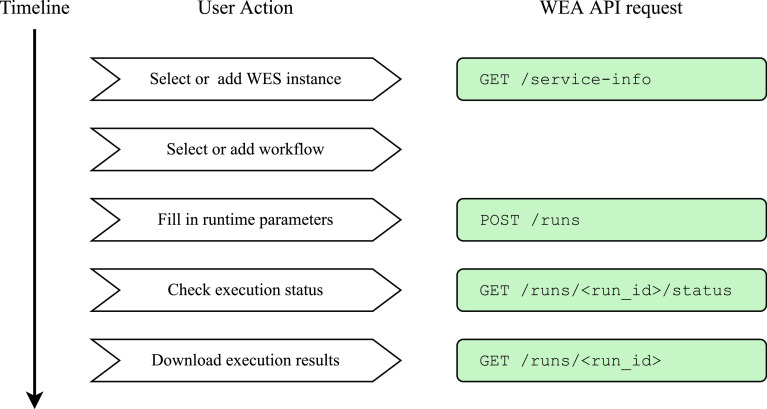
User actions to execute a workflow on Sapporo-web. Sapporo-web provides a step-by-step user interface to help users set up a workflow run. First, users need to specify where to execute a workflow (Workflow Execution Service (WES) instance). Next, users select what to run (workflow) and then how it should be run (input parameters). The UI allows users to download a set of input parameters, which users can upload to re-run a workflow with the same parameters.

**Figure 6.**
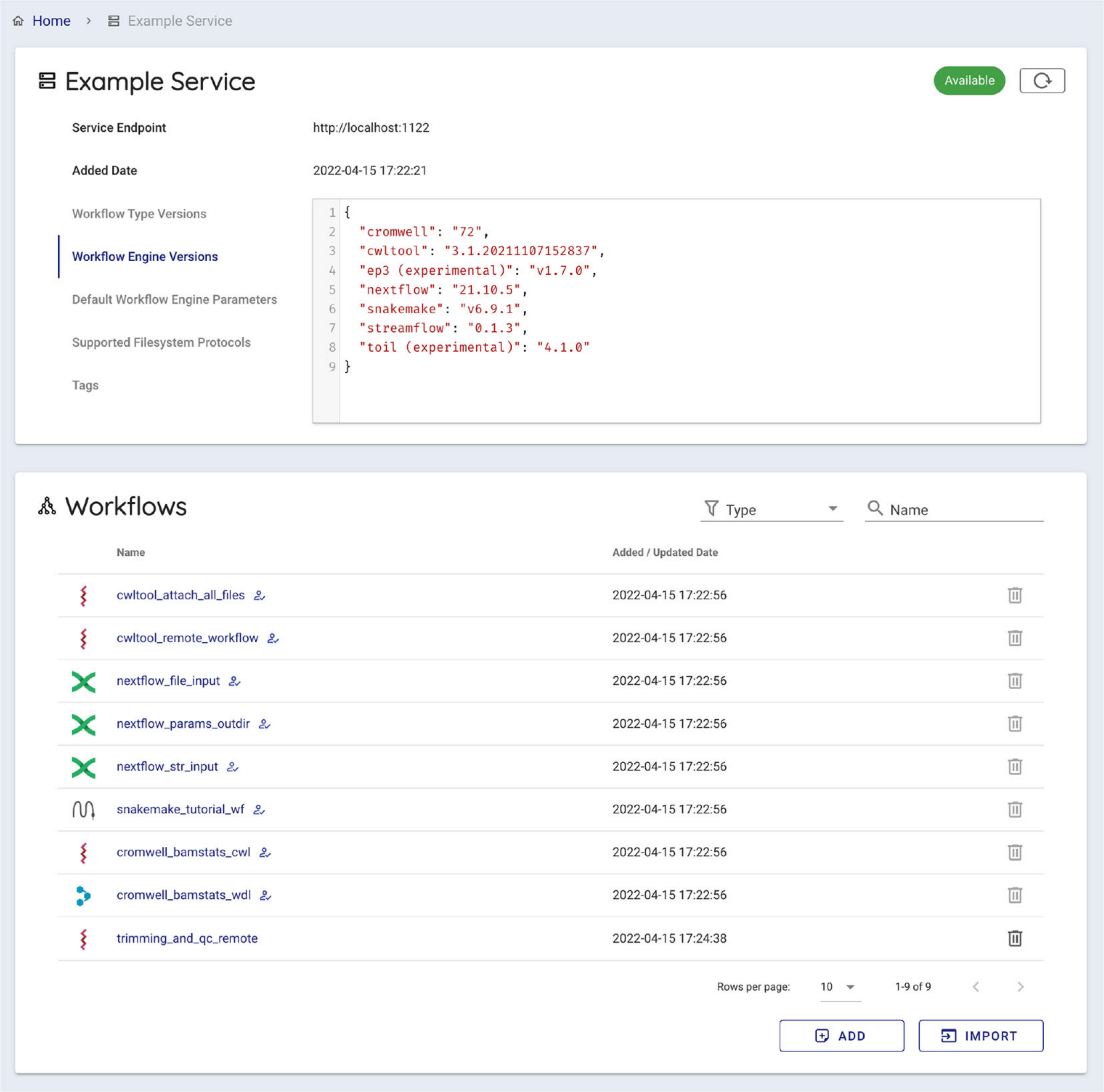
Sapporo-web displays the metadata of the specified Workflow Execution Service (WES) endpoint. The Global Alliance for Genomics and Health (GA4GH) WES specification defines the scheme of the response of service-info. It has the basic information of the WES endpoint, such as supported workflow language and workflow engines. Sapporo-web reads the WES metadata and provides the interface to start composing a workflow run.

**Figure 7.**
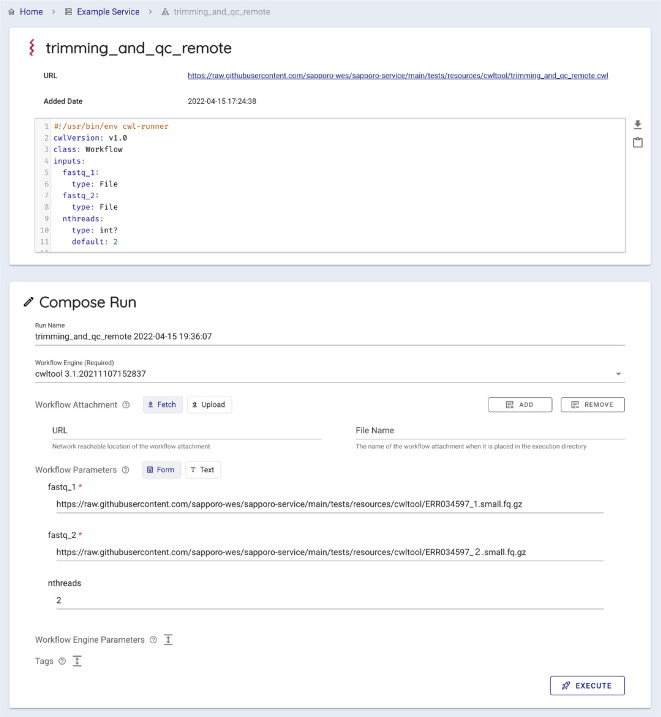
The generated web form to input parameters. Sapporo-web automatically generates a typed input form for a given workflow. It is possible when the given workflow language has a structured job configuration file, for example, a YAML format file for a Common Workflow Language (CWL) workflow. The form has the type validation function for users’ input, such as a file, integer, or array of strings.

To execute a workflow with Sapporo-web, users take the following five steps ( Figure 5). Users can use a WES endpoint either running remotely or locally. Following the user’s connection request, Sapporo-web requests the service-info API of the WES to read the endpoint metadata and display the information ( Figure 6). Users can select a workflow to run by entering a published workflow URL, uploading a workflow definition file, or selecting from the workflows registered on the WES server. Sapporo-web also can accept the GA4GH Tool Registry Service (TRS) protocol as a source of published workflows. Sapporo-web retrieves the content of the requested workflow definition file to generate a web form for entering input parameters ( Figure 7). The type of web form depends on the workflow language. For example, loading a workflow described in Common Workflow Language (CWL) generates a typed input form per parameter because CWL specifies input parameters with a structured text form. ^23^ In contrast, loading a workflow described in languages other than CWL generates a text editor to change the parameters in the corresponding format. After the edit, users can click “execute” to request the workflow to run on the server where the WES endpoint is running.

While the workflow is running, users can check the execution log via Sapporo-web. The standard output and the standard error of the workflow run retrieved from the WES endpoint show up in the log history section. The running status becomes “complete” when the execution finishes on the server. Workflow outputs stored in the WES server are downloadable via a link in the Sapporo-web user interface. If the workflow run failed with an error, the status “executor error” would be shown. Users can visualize the error log in Sapporo-web.

## Results

We developed Sapporo as a WES implementation that allows developers to add new workflow systems. Developers only need to implement the command line procedure in the run.sh script, which is a simple bash script. The project was hosted on GitHub from its inception, with the intention to have other developers contribute with new workflow systems. A good example of this in practice can be seen in the pull request ( https://github.com/sapporo-wes/sapporo-service/pull/29) that added a new workflow system called StreamFlow. ^20^

To evaluate the practical applicability and robustness of Sapporo, we executed the public workflows that researchers frequently use. Specifically, we chose the Mitochondrial Short Variant Discovery workflow from the GATK best practices (written in WDL), the RNA-seq workflow from the nf-core repository (written in Nextflow), and a Germine Short Variant Discovery workflow for processing whole-genome sequencing data from the Japanese Genotype-phonotype Archive (written in CWL). ^24^ Users access Sapporo’s endpoint specifying the input parameters following the WES specification. The required parameters are workflow_url, workflow_type, workflow_type_version, and workflow_params. The workflow_url argument specifies the location of the workflow definition file (e.g. CWL file) to be executed, typically hosted on a remote server, enabling the API to access and utilize the workflow’s instructions. The workflow_params argument points to a JSON file containing input parameters essential for the workflow execution, facilitating customization and adaptation of the workflow’s behavior. The optional arguments workflow_type and workflow_type_version arguments indicate the type and version of the workflow language being employed, ensuring compatibility and proper interpretation of the workflow instructions by engines supported inside Sapporo. Additionally, the workflow_engine_name argument specifies the execution engine to be used, while the default engine for the given workflow language is assigned when it is not specified. Lastly, another optional argument workflow_engine_parameters argument allows for the specification of additional parameters tailored to the execution engine, providing fine-grained control over the execution environment and behavior of the workflow engine. We published the detailed description of the test procedures for these workflows on GitHub, ^25^ and the results of the test runs on Zenodo. ^26^^–^^28^

**Figure 8.**
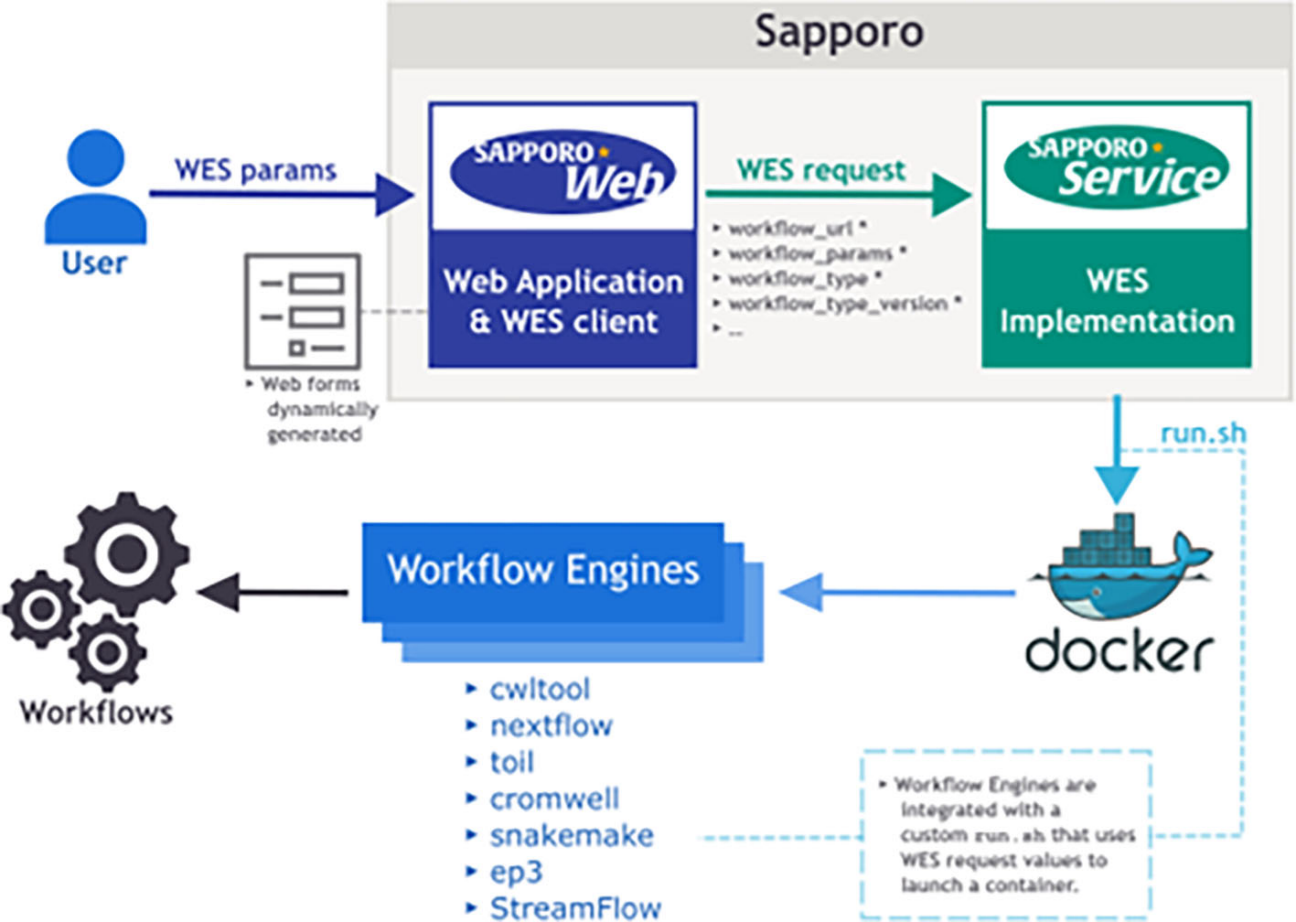
The detailed view of users procedure to run a workflow on Sapporo.

Using a simple CWL workflow as an example, we describe the procedures we performed in the evaluation ( Figure 8). It is noteworthy that despite changes in workflow languages, the steps remain the same, differing only in the supplied workflow definition file or the runtime parameters specified within the designated files. Firstly, the Sapporo-service is initiated within a computational environment. There are two methods for initiating the service: one involves executing a Python program natively, and the other utilizes our Docker image. If Docker or a Docker-compatible Linux container system is available, using the Docker image is simpler. Once the Service is initiated, by default, the API is available via port 1122. The workflow can be executed by sending a POST request to the/runs endpoint of this API. The POST request must include the location of the definition file for the workflow to be executed and the runtime parameters as a part of the URL parameters. Requests to the Sapporo-service can be made by using command-line programs such as curl, scripts written in any programming language, or via our developed web UI, Sapporo-web. Here, we explain the method using curl. Assuming that the Sapporo-service is running on port 1122 of the localhost, the curl command for the request would be as follows:

curl -X POST -F "workflow_url= https://raw.githubusercontent.com/pitagora-network/pitagora-cwl/master/workflows/download-fastq/download-fastq.cwl" -F "workflow_type=CWL" -F "workflow_type_version=v1.0" -F "workflow_engine_name=cwltool" -F "workflow_params=<workflow_params.json" -F "workflow_engine_parameters=<workflow_engine_parameters.json" http://localhost:1122/runs

In this request, a CWL workflow named download-fastq, publicly available on GitHub, is specified. The type of workflow is CWL, with version v1.0, and the workflow engine designated for executing this workflow is cwltool. While there are workflow languages like CWL that can be executed by multiple engines, there are also languages like Nextflow that can only be executed by the nextflow program. Therefore, users must choose the appropriate engine here; otherwise, errors will occur. Information on which engines support which languages can be retrieved via API requests. Parameters to be supplied to the download-fastq workflow for execution via workflow_params, and parameters to be supplied to the workflow engine cwltool via workflow_engine_parameters are specified. Both are described within JSON files and attached to the request as files. Upon receiving this request, the API server issues a UUID to identify this workflow run and returns it as part of the API response. Using this UUID, users can check the status of the run or download results after execution. This API, compliant with GA4GH WES, is straightforward, allowing for the execution of workflows written in various workflow languages within the same computational environment without needing to rewrite the client based on differences in workflow languages.

## Discussion

In the big-data era in biology, the demand for efficient data processing will never stop increasing. ^29^ There are countless painful tasks in data processing, and researchers have developed methods to solve each of them, resulting in many different workflow systems. ^30^ We appreciate that many options are available as open-source so that researchers can choose one for their specific needs. The situation strongly encourages open science: each workflow system community is there so that individuals can help each other by sharing resources. ^31^ However, as each community grows, the gap between the communities also becomes larger. We developed Sapporo to bridge these gaps by providing a new layer to better utilize resources across communities. As the workflow systems are for the increased productivity of data scientists, improving resource interoperability must not interfere with researchers doing their science. An upper layer, the layer of workflow execution, can be a better solution than proposing a new language convertible to other existing languages. The concept of abstraction of workflow execution, as well as the idea of “bringing the algorithms to the data,” is also proposed by the GA4GH cloud work stream, which resulted in the development of the GA4GH Cloud standards. As of May 2022, the GA4GH WES specification supports only CWL and WDL for their workflow format. There is no official list of WES implementations; however, no other service that allows the addition of workflow systems is available as far as we investigated.

To support workflow developers and researchers conducting data analysis, multiple different workflow management systems have been developed. These systems enhance productivity and reproducibility in data analysis, enabling more effective science. However, the proliferation of multiple systems has revealed inefficiencies, leading to fragmentation within developer and user communities. While it’s crucial to effectively leverage the assets of each system and community, it is not practical to provide the methods for syntax conversion between workflow systems and extending execution engines. Therefore, Sapporo aims to absorb differences between systems by wrapping multiple systems. Specifically, we provide an API that rewrites workflow definitions and runtime parameters into the command lines of each system based on the type of workflow definition received, enabling the execution of different workflows using the same client. The Web API adheres to the internationally defined GA4GH WES standard, ensuring interoperability with other GA4GH WES implementations. By developing and releasing Sapporo Web as an example of a GA4GH WES client, we demonstrate the readiness of our developed API for research use.

Although Sapporo is a flexible system covering many use cases, we recognize that the current implementation has a few technical limitations. The main objective of Sapporo is to absorb the variance of the execution methods per workflow system. We achieved building a unified way to request a workflow run by providing the API and its client. However, there is still a challenge in the user experience with regard to the parameter editing function. This is caused by differences in the workflow system concepts. For example, some workflow systems, such as Nextflow or Snakemake, use Domain Specific Language (DSL) model in their syntax for better productivity, so users can write a workflow as they would write a script in their preferred programming language. ^9^^,^^18^ However, this flexibility in describing the procedures often makes the required input parameters unparsable by other applications. It means that users need to learn how to edit the parameters for each workflow system they are using. Though often this is not too difficult, the workflow system communities need to lower the learning costs to use a workflow. For example, finding a more generic representation of workflow inputs between workflow language systems could alleviate the situation.

Sapporo is a unique WES implementation that accepts multiple workflow languages. Researchers can use the system to utilize community workflows without regarding what language they are written in. One downside of this flexibility is that errors reported by Sapporo from different workflow engines may not look familiar to users. Many well-maintained workflow registries are available, such as nf-core and WorkflowHub, but the quality of the workflows published in these registries relies on each community’s efforts. ^10^^,^^32^^,^^33^ A system that validates and verifies the quality of workflows is also required for the sustainability of the resources published in the workflow registries.

Data processing methods vary greatly depending on the type of input data and the computational platform. In bioinformatics, the laboratory equipment and computers available drive changes. New computing applications for efficient data science, and new problems of resource portability may appear if variables such as input data, equipment, and computing resources keep changing in the future. Through its concept of abstraction, Sapporo can be a key player in assisting different communities in sharing and reusing workflows and other computing resources.

## Data availability

All of these projects are licensed under the Apache License 2.0.

### Underlying data

Zenodo: sapporo-wes/test-workflow: 1.0.1. https://doi.org/10.5281/zenodo.6618935. ^25^

This project contains the following underlying data:

• sapporo-wes/test-workflow-1.0.1.zip (description of the test procedures and results of the workflows described in section *Use cases*).

The results of the test runs are contained in the following projects:

• Zenodo: Sapporo execution results - broadinstitute/gatk/MitochondriaPipeline: 1.0.0. https://doi.org/10.5281/zenodo.6535083. ^26^
• Zenodo: Sapporo execution results - nf-core/rnaseq: 1.0.0. https://doi.org/10.5281/zenodo.6534202. ^27^
• Zenodo: Sapporo execution results - JGA analysis - per-sample: 1.0.0. https://doi.org/10.5281/zenodo.6612737. ^28^

### Extended data

Zenodo: sapporo-wes/sapporo: 1.0.0. https://doi.org/10.5281/zenodo.6462774. ^34^

This project contains the following extended data:

• Sapporo: Getting Started.md (step-by-step procedures for deploying a Sapporo instance on a local computer and testing the system).

## Software availability

Sapporo-service’s source code, test code, and documentation:

• Source code available from: https://github.com/sapporo-wes/sapporo-service/tree/1.2.4
• Archived source code at time of publication: https://doi.org/10.5281/zenodo.6609570. ^35^
• License: Apache License 2.0

Sapporo-web’s source code, test code, and documentation:

• Source code available from: https://github.com/sapporo-wes/sapporo-web/tree/1.1.2
• Archived source code at time of publication: https://doi.org/10.5281/zenodo.6462809. ^36^
• License: Apache License 2.0
