# Supplementary material for: Sapporo: A workflow execution service that encourages the reuse of workflows in various languages in bioinformatics
Source: F1000Res. 2024 Jun 24;11:889. Originally published 2022 Aug 4. [Version 2] doi: 10.12688/f1000research.122924.2 (PMC11282396; doi:10.12688/f1000research.122924.2)
Supplement: Peer review history

# Reviewer report: Reviewer response for version 2

**DOI:** 10.5256/f1000research.167905.r294852

**Published:** 2024-10-30

**Authors:** Iacopo Colonnelli

**Affiliations:** 1 Universita degli Studi di Torino, Turin, Piedmont, Italy

The author successfully addresses most of the concerns affecting the first version of this work. For the remaining ones, they provided exhaustive and convincing justifications in the comments. The article is now mature enough to be indexed.

Are the conclusions about the tool and its performance adequately supported by the findings presented in the article?

Partly

Is the rationale for developing the new software tool clearly explained?

Yes

Is the description of the software tool technically sound?

Yes

Are sufficient details of the code, methods and analysis (if applicable) provided to allow replication of the software development and its use by others?

Partly

Is sufficient information provided to allow interpretation of the expected output datasets and any results generated using the tool?

No

Reviewer Expertise:

Workflows, High Performance Computing, Parallel Computing, Distributed Computing

I confirm that I have read this submission and believe that I have an appropriate level of expertise to confirm that it is of an acceptable scientific standard.

# Reviewer report: Reviewer response for version 2

**DOI:** 10.5256/f1000research.167905.r305477

**Published:** 2024-07-27

**Authors:** Stephen R. Piccolo

**Affiliations:** 1 Department of Biology, Brigham Young University, Provo, UT, USA

This paper describes the Sapporo system, which is designed to be a Web-based wrapper around existing workflow engines. It allows users to specify workflows to be executed on various different engines on the back end. It is compatible with the GA4GH WES standard. The paper clearly describes motivations behind the software's existence, how a researcher might use it, and limitations of the software. The software is open source, and examples of its use are provided. Personally, I am unsure whether I would use the software because it adds a layer of complexity. I would probably just use the underlying workflow engine if I already had a workflow file. Then again, if I typically used one particular workflow engine and found a better workflow in a different ecosystem, I might use Sapporo to facilitate execution. On the other hand, a challenge is that I would likely need to specify workflow_engine_parameters values that are specific that new workflow engine. That would take some time to figure out, so maybe I would just use the underlying workflow engine after all. Having said this, I think it is good that the software exists so that it is an option for researchers to use.

 I have one more question/comment. CWL workflows can execute tasks within Docker. (I forget which other workflow systems support this.) However, the Sapporo system also uses Docker. It is tricky to run Docker from within Docker. So I am unsure how that situation is handled.

Are the conclusions about the tool and its performance adequately supported by the findings presented in the article?

Yes

Is the rationale for developing the new software tool clearly explained?

Yes

Is the description of the software tool technically sound?

Yes

Are sufficient details of the code, methods and analysis (if applicable) provided to allow replication of the software development and its use by others?

Yes

Is sufficient information provided to allow interpretation of the expected output datasets and any results generated using the tool?

Yes

Reviewer Expertise:

Bioinformatics, genomics, workflow management systems, education

I confirm that I have read this submission and believe that I have an appropriate level of expertise to confirm that it is of an acceptable scientific standard, however I have significant reservations, as outlined above.

# Reviewer report: Reviewer response for version 2

**DOI:** 10.5256/f1000research.167905.r305473

**Published:** 2024-07-26

**Authors:** Denis Yuen

**Affiliations:** 1 Ontario Institute for Cancer Research, Toronto, Ontario, Canada

This article largely covers the technical design decisions behind Sapporo, a suite of tools for facilitating workflow execution, while focusing on sapporo-service and sapporo-web. 

 Overall, the authors should be commended for their efforts in aiding reproducible science by making it simpler to run workflows using multiple workflow languages. Their efforts also sand-off the rough edges of the GA4GH workflow execution service (WES) standard making it easier for the community as a whole to develop tools to run workflows in a simpler uniform manner. Efforts to write standards and aid reproducible science can often be unsung but are deeply valuable to science as a whole.

 Under the header of data and software availability, both GitHub repositories are well laid out and I was able to easily use docker-compose to spin up both components for some simple sanity checks. I also commend the authors for following best practices including well developed documentation for developers, continuous integration, DOIs on Zenodo, and last but not least regular software releases conforming to semantic versioning. 

 As for the manuscript, I recommend that the 2024 revision of the manuscript be accepted with only a couple minor suggestions for clarification. 

 There is one reference to 2022 in the discussion, “As of May 2022, the GA4GH WES specification supports only CWL and WDL for their workflow format.” Since this revision is in 2024 and In light of the 1.1.0 release of the standard on Sept 14, 2023 it may be useful to update. For example, in  table 2, it is clear Sapporo supports more than these two languages yet is built on top of WES in figure 2. Has the WES standard been expanded to more languages or did Sapporo need to make extensions to the standard?

 Also in table 2, it should be noted that Toil has WDL support although this may/may not be supported by Sapporo.

Are the conclusions about the tool and its performance adequately supported by the findings presented in the article?

Yes

Is the rationale for developing the new software tool clearly explained?

Yes

Is the description of the software tool technically sound?

Yes

Are sufficient details of the code, methods and analysis (if applicable) provided to allow replication of the software development and its use by others?

Yes

Is sufficient information provided to allow interpretation of the expected output datasets and any results generated using the tool?

Yes

Reviewer Expertise:

scientific workflows, cloud computing

I confirm that I have read this submission and believe that I have an appropriate level of expertise to confirm that it is of an acceptable scientific standard.

# Reviewer report: Reviewer response for version 1

**DOI:** 10.5256/f1000research.134975.r185836

**Published:** 2023-10-05

**Authors:** Iacopo Colonnelli

**Affiliations:** 1 Universita degli Studi di Torino, Turin, Piedmont, Italy

In this article, the authors describe Sapporo, a WES-compatible web-based interface to multiple workflow management systems (WMSs). Sapporo aims to act as a common interface to different underlying WMSs, abstracting product-specific details and complexities to the end users. This way, Sapporo fosters WMSs interoperability and lowers the technical barriers between domain experts and workflow execution.

 The description of Sapporo is kept at a high level of abstraction, without many technical details about the implementation. However, it is detailed enough to let the reader capture all the crucial aspects of the software architecture, the frontend and backend structure, and the main limitations of the current version. A link to a Docker Compose manifest for quick evaluation would be a plus.

 For implementers, the description is too high-level to understand how to replicate the software development. Additional material published on GitHub and Zenodo contains further details for the developers. However, having at least a high-level description of the `run.sh` script, which constitutes the core of the Sapporo backend, would improve the article's understandability.

 The main flaw of the article in its current form is that the description of the experimental evaluation and the obtained results is left to external references. The authors describe three different experiments using three different workflow languages and datasets. Adding a detailed description of one of them directly in the article, together with the steps needed to reproduce it, would allow the reader to better understand the features Sapporo provided.

 Also, there is no quantitative measure of achieved results in the article. Some quantitative measures of the Sapporo complexity (e.g., the percentage of product-specific lines of code that users are still forced to write to specify workflow parameters or the average lines of code needed to add support for a new WMS) would enable a more scientifically sound evaluation of the product.

Are the conclusions about the tool and its performance adequately supported by the findings presented in the article?

Partly

Is the rationale for developing the new software tool clearly explained?

Yes

Is the description of the software tool technically sound?

Yes

Are sufficient details of the code, methods and analysis (if applicable) provided to allow replication of the software development and its use by others?

Partly

Is sufficient information provided to allow interpretation of the expected output datasets and any results generated using the tool?

No

Reviewer Expertise:

Workflows, High Performance Computing, Parallel Computing, Distributed Computing

I confirm that I have read this submission and believe that I have an appropriate level of expertise to confirm that it is of an acceptable scientific standard, however I have significant reservations, as outlined above.

# Response

**Published:** 2024-06-13

**Authors:** Tazro Ohta

**Affiliations:** DBCLS, Japan

We deeply thank you for taking the time to review our manuscript.

***1. A link to a Docker Compose manifest for quick evaluation would be a plus.***

 Thank you for the great suggestion. We added a line to indicate the location of the docker compose manifest for each of Sapporo-service and Sapporo-web.

***2. For implementers, the description is too high-level to understand how to replicate the software development. Additional material published on GitHub and Zenodo contains further details for the developers. However, having at least a high-level description of the `run.sh` script, which constitutes the core of the Sapporo backend, would improve the article's understandability.***

 We agree with your assessment. We modified the paragraphs in the subsection "Workflow execution service" in the Methods section to describe the run.sh function of Sapporo-service:

*> The system is designed to separate the execution layer from the handling of API requests, thereby enhancing modularity and extensibility. The execution layer operates through a well-structured shell script named "run.sh." Upon receiving an API request, the system forks "run.sh," which then generates command lines for the workflow system and executes them. This separation enables the addition of new workflow systems without changes to the API server's code. As a result, adding new workflows becomes straightforward, with the number of systems growing from just one at the beginning of the project to seven in the current version (Table 2). The flexibility of the "run.sh" also allows for specific adjustments for each workflow system, supporting pre- and post-execution processes, such as authentication, staging input files, and uploading results. Additionally, it is enabled to manage environment-specific requirements, including executing jobs on grid engines and handling file I/O with S3-like object storage. Once the system receives a workflow run request, it issues a universally unique identifier (UUID) and creates a directory named with the UUID, where the system stores all the necessary files. The workflow definition files, intermediate and final outputs, and the other metadata are stored in that directory. This per-run directory can act as a bundle of provenance for the workflow run (Figure 4).*

***3. The main flaw of the article in its current form is that the description of the experimental evaluation and the obtained results is left to external references. The authors describe three different experiments using three different workflow languages and datasets. Adding a detailed description of one of them directly in the article, together with the steps needed to reproduce it, would allow the reader to better understand the features Sapporo provided.***

 In response to the comment by another reviewer (Dr. Justin M. Wozniak), we added the details of how users can specify the workflow condition in the Result section and the new figure Figure 8. We believe the addition can guide users to understand how they can run a workflow using our implementation.

***4. Also, there is no quantitative measure of achieved results in the article. Some quantitative measures of the Sapporo complexity (e.g., the percentage of product-specific lines of code that users are still forced to write to specify workflow parameters or the average lines of code needed to add support for a new WMS) would enable a more scientifically sound evaluation of the product.***

 We agree with the reviewer's feedback. Indeed, quantitatively evaluating how much the adoption of our system reduces the barrier to using different workflow management systems is challenging, which made this article posted as a software tool article. Methods such as user surveys could be considered for evaluation, but attempting to familiarize participants with multiple workflow systems without the assistance of our system or having learners of one language use a system in another language would not be practical. Demonstrating quantitatively that "using workflow systems is inherently more productive than executing workflows built with shell scripts on job queuing systems via the command line" poses a challenge for the entire developer community involved in developing workflow systems. Considering the challenges in the evaluation, with this paper we believe that providing the option of not only multiple different workflow management systems but also a system that can be used across them is our main contribution to the community.

# Reviewer report: Reviewer response for version 1

**DOI:** 10.5256/f1000research.134975.r185842

**Published:** 2023-07-26

**Authors:** Justin M. Wozniak

**Affiliations:** 1 Data Science and Learning, Argonne National Laboratory, Lemont, IL, USA

This article presents a new abstraction layer (Sapporo) over existing workflow systems to support portability and interoperability. The paper is oriented around a bioinformatics workload. The paper is primarily focused on how Sapporo operates as a web service and runs underlying workflows using the other existing systems.

 The paper provides a pretty good high-level state-of-the-art in the workflow ecosystem and the bioinformatics use case.

 The paper spends most of its space describing the abstraction over workflow systems and figures that illustrate the corresponding architecture. It does not contain a deep dive into any challenges regarding workflow system interoperability.

 From a bioinformatics perspective, the description of support for the application workload is very high-level. There is no deep dive into what is really required to make this workload work. There are results posted for the run that are linked on the Internet, but they are not summarized in the text.

 The architecture figures are very spacious and do not provide much technical insight.

 There is an illustration of the web form used to run Sapporo, but it seems oversimplified and does not convey what the user would be faced with in a real-world problem.

 I downloaded the Sapporo source zip via the links in the paper. The source tree was not very revealing and I am not set up to run a new web service. The README pointed me to web-based docs that seemed quite good and included detailed installation notes.

 Overall, this is a good high-level introduction to Sapporo, but does not offer detailed technical insights into the problem space or the Sapporo solution. More details are necessary about these topics to provide insight to the community and allow for a better evaluation of the Sapporo contribution.

Are the conclusions about the tool and its performance adequately supported by the findings presented in the article?

Partly

Is the rationale for developing the new software tool clearly explained?

Yes

Is the description of the software tool technically sound?

Partly

Are sufficient details of the code, methods and analysis (if applicable) provided to allow replication of the software development and its use by others?

No

Is sufficient information provided to allow interpretation of the expected output datasets and any results generated using the tool?

No

Reviewer Expertise:

workflows, hpc, distributed computing

I confirm that I have read this submission and believe that I have an appropriate level of expertise to confirm that it is of an acceptable scientific standard, however I have significant reservations, as outlined above.

# Response

**Published:** 2024-06-13

**Authors:** Tazro Ohta

**Affiliations:** DBCLS, Japan

We deeply thank you for taking the time to review our manuscript. 

***1. The paper spends most of its space describing the abstraction over workflow systems and figures that illustrate the corresponding architecture. It does not contain a deep dive into any challenges regarding workflow system interoperability.***

 We agree with your assessment. We added the text below in the Background section to emphasize the challenge in making the existing workflow systems:

*> Workflow systems have different language syntaxes and engines, each designed for specific purposes. For instance, Nextflow aims to boost developer productivity and scalability, while Snakemake focuses on flexibility and simplicity, using Python as its base. In contrast, the Common Workflow Language (CWL) project aims to promote interoperability by creating a standardized syntax that various workflow engines can understand. However, workflows written in different languages cannot be easily converted into each other automatically. The most popular workflow systems used in bioinformatics, such as CWL, WDL, Nextflow, and Snakemake, take a workflow definition and input parameters to produce output result files, while there are differences between these workflow systems in command-line options, workflow description syntax, methods for specifying inputs, and how expected output files are defined.*

*> Creating a universal language converter isn't practical because some languages lack the necessary syntax parsers, or contain features that are not commonly found in other workflow engines (e.g. JavaScript evaluation as in CWL, loops in workflows or cyclic workflows instead of DAG-based systems). To bridge the gap between different workflow systems, we need a standardized way to specify workflows, input parameters, and expected outputs. Additionally, a system that supports various engines and selects the appropriate one for a given workflow is essential for smooth interoperability.*

***2. From a bioinformatics perspective, the description of support for the application workload is very high-level. There is no deep dive into what is really required to make this workload work. There are results posted for the run that are linked on the Internet, but they are not summarized in the text.***

 We agree again with your assessment. To clarify the user's procedure for performing the analysis, we modified the paragraph in the Result section as follows:

*> To evaluate the practical applicability and robustness of Sapporo, we executed public workflows frequently used by researchers. Specifically, we chose the Mitochondrial Short Variant Discovery workflow from the GATK best practices (written in WDL), the RNA-seq workflow from the nf-core repository (written in Nextflow), and a Germine Short Variant Discovery workflow for processing whole-genome sequencing data from the Japanese Genotype-phonotype Archive (written in CWL). Users access Sapporo's endpoint specifying the input parameters following the WES specification. The required parameters are workflow_url, workflow_type, workflow_type_version, and workflow_params. The workflow_url argument specifies the location of the workflow definition file (e.g. CWL file) to be executed, typically hosted on a remote server, enabling the API to access and utilize the workflow's instructions. The workflow_params argument points to a JSON file containing input parameters essential for the workflow execution, facilitating customization and adaptation of the workflow's behavior. The arguments workflow_type and workflow_type_version arguments indicate the type and version of the workflow language being employed, ensuring compatibility and proper interpretation of the workflow instructions by engines supported inside Sapporo. Additionally, the workflow_engine_name argument specifies the execution engine to be used, while the default engine for the given workflow language is assigned when it is not specified. Lastly, another optional argument workflow_engine_parameters argument allows for the specification of additional parameters tailored to the execution engine, providing fine-grained control over the execution environment and behavior of the workflow engine.  We published the detailed description of the test procedures for these workflows on GitHub, and the results of the test runs on Zenodo.*

 We also added a detailed view of user's procedure to run a workflow with Sapporo as Figure 8.

***3. The architecture figures are very spacious and do not provide much technical insight.***

 The architecture figures presented in the paper aim to illustrate the concept of our approach, which is not a monolithic software but rather divided into multiple layers. The figure aims to focus on the concept itself, because the technologies employed for implementation may change in the future. While we implemented the Sapporo-service providing the Web API in Python Flask and the Web UI accessing the API in Vue.js to enhance our development efficiency, we do not claim technical superiority over them here. As the software stacks evolve, we acknowledge the possibility of changing technology choices in the future. The idea of decomposing components into functionalities, as exemplified by microservices architecture, is common in today's software engineering, and we do not consider it novel. However, in the field of bioinformatics where existing workflow systems tend to be monolithic, easily extensible and layered systems are not as prevalent. Thus, we argue in the paper that such a design is beneficial for solving the problem we raise. Nonetheless, from the series of comments received from the reviewer, we recognized that the main message of the paper might not have been as clear as we intended. We believe the paragraphs added in Background and Discussion according to your comments may help readers to understand the aim of our projects.

***4. There is an illustration of the web form used to run Sapporo, but it seems oversimplified and does not convey what the user would be faced with in a real-world problem.***

 We understand the reviewer's concern about the oversimplified depiction of the workflow in Figure 7, resulting in a very basic UI representation that may differ from what users may encounter when using Sapporo-web in real-world scenarios. The intention behind this illustration was to demonstrate the automatic rendering of the UI based on the input in the workflow definition file. We do not assume or claim here that highly complex workflows with complicated input parameter sets can be executed seamlessly through the generated Web UI. We added the following sentences in the Discussion section to claim the relationship between the user interface and the improvement of workflow usability:

*> As the complexity of the workflow increases, so does the difficulty of executing it. Sapporo's primary goal is to reduce the time and learning costs associated with deployment, parameterization, and execution due to changes in workflow languages or systems, not to reduce the inherent complexity of given workflows. The use of complex workflows requires complex input parameter specifications due to the intricacies of their internal processes, and using them without understanding them would not reflect scientific integrity in data analysis. Of course, some costs can be mitigated through UI enhancements, such as optimizing sets of multiple input parameters or streamlining iterations for numerous input files. However, it's not practical for Sapporo-web's default UI to cover all these scenarios, as web UIs are not one-size-fits-all. There is potential to solve this problem by semi-automatically generating a UI for each workflow, a concept we're exploring in another project. However, even in this scenario, the advantage of splitting our UI into standardized APIs remains apparent.*

***5. Overall, this is a good high-level introduction to Sapporo, but does not offer detailed technical insights into the problem space or the Sapporo solution. More details are necessary about these topics to provide insight to the community and allow for a better evaluation of the Sapporo contribution.***

 In response to your comment; we believe that your previous points stressed this same point, and the content added there addresses this same issue. As outlined in our response to your first comment, we have added descriptions in the updated manuscript about the issues Sapporo aims to address. Additionally, we have incorporated the following passage into the Discussion section:

*> To support workflow developers and researchers conducting data analysis, multiple different workflow management systems have been developed. These systems enhance productivity and reproducibility in data analysis, enabling more effective science. However, the proliferation of multiple systems has revealed inefficiencies, leading to fragmentation within developer and user communities. While it is crucial to effectively leverage the assets of each system and community, it is not practical to provide the methods for syntax conversion between workflow systems and extending execution engines. Therefore, Sapporo aims to absorb differences between systems by wrapping multiple systems. Specifically, we provide an API that rewrites workflow definitions and runtime parameters into the command lines of each system based on the type of workflow definition received, enabling the execution of different workflows using the same client.  Inside the API server, we use Docker containers to ensure the usability of different workflow engines. The use of containers also ensures future additions of workflow engines while maintaining the portability of the API server. The Web API adheres to the internationally defined GA4GH WES standard, ensuring interoperability with other GA4GH WES implementations. By developing and releasing Sapporo Web as an example of a GA4GH WES client, we demonstrate the readiness of our developed API for research use.*
